# GPX4 activator enhances neuroprotection and functional recovery in spinal cord injury

**DOI:** 10.1016/j.jot.2025.03.013

**Published:** 2025-05-07

**Authors:** Xinjie Liu, Yilin Pang, Baoyou Fan, Jiawei Zhang, Shen Liu, Xiaobing Deng, Yun Li, Ying Liu, Xu Zhang, Chenxi Zhao, Xiaoyu Wang, Xudong Wu, Luhua Lai, Shiqing Feng, Wenpeng Liu, Guangzhi Ning, Xue Yao

**Affiliations:** a Tianjin Key Laboratory of Spine and Spinal Cord, Tianjin Institute of Orthopeadic Innovation and Translation, International Science and Technology Cooperation Base of Spinal Cord Injury, Department of Orthopedics, Tianjin Medical University General Hospital, International Chinese Musculoskeletal Research Society Collaborating Center for Spinal Cord Injury, Tianjin, 300052, China; b Department of Orthopedics, Qilu Hospital of Shandong University, Shandong University Centre for Orthopedics, Advanced Medical Research Institute, Shandong University, Jinan, Shandong, 250012, China; c College of Chemistry and Molecular Engineering, Peking University, Beijing, 100871, China; d Tianjin Key Laboratory of Metabolic Diseases, Department of Physiology and Pathophysiology, The Province and Ministry Co-sponsored Collaborative Innovation Center for Medical Epigenetics, Center for Cardiovascular Diseases, Research Center of Basic Medical Sciences, Tianjin Medical University, Tianjin, 300070, China; e Department of Cell Biology, School of Basic Medical Sciences, Tianjin Medical University, Tianjin, 300070, China; f Renal Division and Division of Engineering in Medicine, Department of Medicine, Brigham Women's Hospital, Harvard Medical School, Harvard University, Boston, MA, 02115, USA

**Keywords:** Spinal cord injury, Ferroptosis, GPX4 activation, Neuroprotection, Neuroinflammation

## Abstract

**Background:**

Spinal cord injury (SCI) exerts severe physical, social, and economic effects on individuals and the healthcare system. While much progress has been made in understanding the pathophysiology of SCI, the regulation of the ferroptosis master regulator, GPX4 (Glutathione Peroxidase 4), remains poorly understood.

**Methods:**

In a rat T10 contusion SCI model, GPX4 expression was tracked with western blot and immunofluorescence. Ferroptosis was induced in primary neurons using the GPX4 inhibitor RSL3, and inflammatory cytokine release was measured. Conditioned media from these neurons was applied to microglia to assess activation. The GPX4 activator PKUMDL-LC-102 was administered to SCI rats, with functional recovery evaluated through behavioral tests, MRI, and motor-evoked potentials.

**Results:**

We first reveal a temporal and spatial decrease of GPX4 levels in neurons after SCI. We then demonstrate that GPX4 inhibition leads to primary neuronal ferroptosis, triggering the secretion of pro-inflammatory cytokines that activate microglia. This study represents the initial *in vivo* investigation of GPX4-specific targeted activation, demonstrating its potential to promote functional recovery in contusive SCI by improving neuronal survival and reducing microgliosis.

**Conclusion:**

These findings highlight the significance of GPX4 as a key factor for neuroprotection in the spinal cord. We identified the pivotal role of GPX4 in SCI and realize the neuroprotection via specific GPX4 activation to improve functional recovery *in vivo*.

**The translational potential of this article:**

These findings provide a novel avenue for therapeutic intervention to enhance functional recovery after SCI through GPX4 targeted activation.

## Graphical abstract

**Figure:**
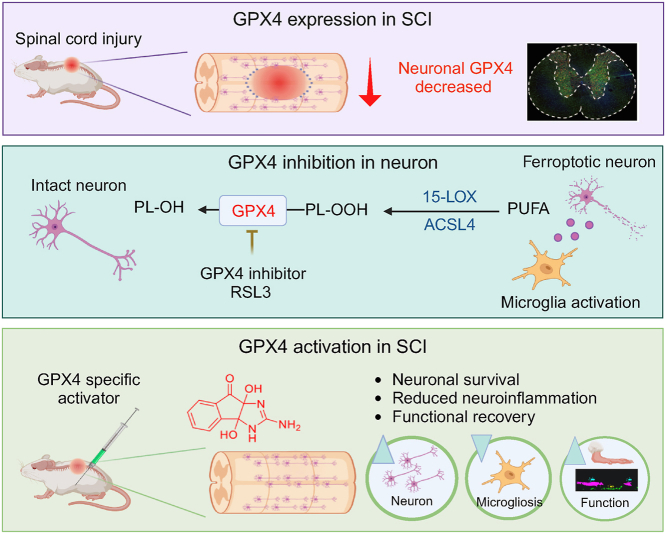

## 1 Introduction

Spinal cord injury (SCI) leads to the loss of sensory and motor function below the injury level, accompanied by various complications, resulting in a significant social and familial burden [[1], [2], [3]]. For example, there were 760,000 prevalent traumatic SCI patients in total and 66,000 new traumatic SCI cases annually in China [4], and the annual incidence of SCI in the United States reaches up to around 17,000 people with estimated lifetime costs exceeding $4.5 million [5]. The pathological processes encompass both primary and secondary injuries [6,7]. Besides, numerous secondary injury events, including the inflammatory cascade, reactive oxygen species (ROS) regeneration, and lipid peroxidation, exacerbate the damage of spinal cord tissue [8,9].

Ferroptosis, characterized by iron-dependent lipid peroxidation [10,11], emerges as a crucial pathophysiological factor in both SCI and other central nervous system (CNS) diseases [[12], [13], [14], [15], [16]]. Glutathione Peroxidase 4 (GPX4), an enzyme containing selenium that acts as a regulator of ferroptosis, detoxifies a wide range of substrates, including hydroperoxide and complex hydroperoxides such as phospholipid hydroperoxides (PLOOH) [17]. GPX4 is also recognized for directly converting complex phospholipid hydroperoxides [18,19]. Depletion of GPX4 results in a notable increase in lipid peroxidation and the induction of ferroptosis [20], and GPX4 deficiency leads to kidney and lung dysfunction [21,22]. In the CNS, GPX4 is critical for neuronal survival and can trigger ferroptosis when its activity is reduced [23,24]. Conditional GPX4 knockout in forebrain neurons leads to neuronal ferroptosis and neurodegeneration, as observed in Alzheimer's disease [25]. GPX4's role in inflammation and immunity is also prominent [26,27]. For instance, GPX4 depletion can activate the cGAS-STING pathway and induce the production of IFN-1 in innate immunity [28]. Moreover, using a small molecule GPX4 activator elevates GPX4 enzyme activity, inhibiting the NF-κB pathway *in vitro* [26]. Recent investigations have demonstrated a decrease in GPX4 levels at 2 days and 7 days after SCI [29]. However, our understanding of the spatial and temporal expression pattern of GPX4 after SCI remains limited, and the effectiveness of GPX4 activation in promoting function recovery after SCI is yet to be determined.

In this study, we implemented the GPX4-targeted approach to promote functional recovery in SCI via neuroprotection *in vivo*. We investigated the temporal and spatial protein expression pattern of GPX4 and explored its role in SCI. Our research revealed a temporal and spatial decrease of GPX4 protein levels in neurons after SCI. Additionally, GPX4 inactivation by RSL3, one type of the GPX4 inhibitor, resulted in ferroptosis and the expression pro-inflammatory cytokines in primary neurons. We also observed the subsequent microglial activation both *in vivo* and *in vitro*. Furthermore, we realized the improved functional restoration following SCI by activating GPX4. These findings suggest that GPX4 activation has significant potential as a therapeutic approach for managing SCI.

## 2 Results

### 2.1 GPX4 expression is downregulated in the acute phase of SCI

We first investigated the temporal dynamics of GPX4 protein expression within spinal cord tissue at different time points after SCI using a contusion model (Fig. 1A). GAPDH was employed as the internal control. The Western blot results revealed that there was no significant change in GPX4 expression at the epicenter of the spinal cord at the 4-h time point following SCI when compared to the Sham-operated group (Fig. 1B). However, at 1, 3, and 7 days following SCI, GPX4 expression exhibited a significant decrease. At 2 week and 3 week the expression level of GPX4 recovered to the same as Sham group. So the GPX4 protein expression showed a particularly prominent reduction during the acute phases of SCI.

**Fig. 1:**
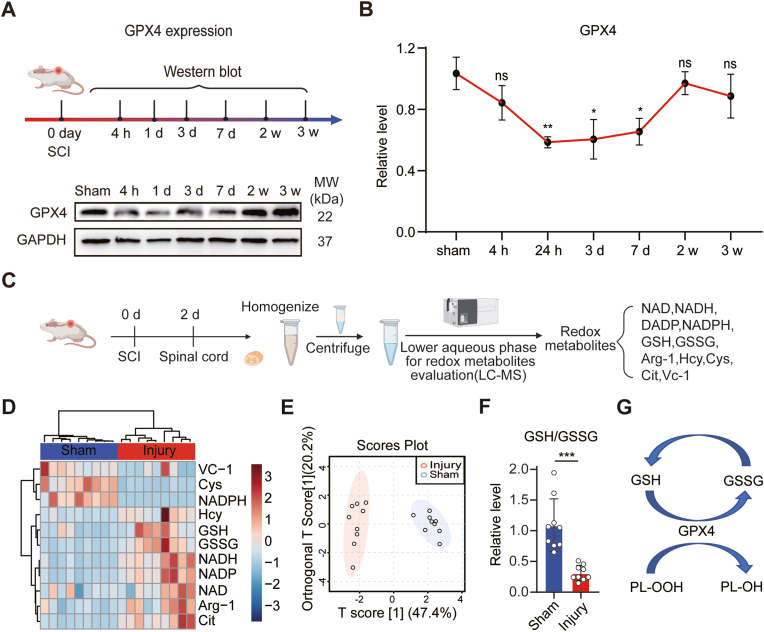
**Temporal expression profile of GPX4 and redox metabolomics after SCI A** Representative Western Blot images of GPX4 at different time points after SCI. **B** Quantification of the GPX4 expression level at different time points after SCI, GAPDH was used as an internal control for Western blot normalization (n = 4, Data are presented as Mean ± SD, and statistical significance was determined by one-way ANOVA with Tukey's post-hoc test). **C** Experimental design of redox environment metabolomic evaluation. **D** Heatmap showing the changing metabolites between Sham and Injury groups at 2 days after SCI. **E** Orthogonal partial least-squares discriminant analysis of redox metabolites (n = 9). **F** GSH/GSSG level at 2 days after SCI (n = 9, Data are presented as Mean ± SD, and statistical significance was determined by unpaired T-TEST). Values are normalized with the sham group. Data were analyzed on MetaboAnalyst 5.0 (https://www.metaboanalyst.ca/). **G** Mechanism of GPX4 inactivation. ∗: p < 0.05, ∗∗: p < 0.01, ns: no significance.

### 2.2 Redox metabolism reveals GSH/GSSG reduction in acute SCI

In order to investigate the impact of GPX4 reduction on the redox metabolism microenvironment in the acute phase of SCI, we conducted a LC-MS/MS-based metabolomic evaluation 2 days after SCI (Fig. 1C). This timing was chosen because ferroptosis predominantly occurs in the acute phase, and GPX4 plays a crucial role in inhibiting this process. We revealed changes of 11 key redox molecules, including redox couples and other related components (Fig. 1D). Orthogonal Partial Least Squares-Discriminant Analysis (orthoPLS-DA) was used for the metabolic profiling (Fig. 1E), revealing distinct clustering of spinal cord tissues from Sham and Injury groups. The clustering pattern indicates significant alterations in the redox profile of spinal cord tissue in the acute phase following SCI compared to the control Sham group. Our analysis reveals that Injury groups possess a significant reduction in the GSH/GSSG ratio compared with Sham Group (Fig. 1F), which is one of the major redox couples within cells and modulated by GPX4. GPX4 expression level in the spinal cord leads to GSH/GSSG ratio decrease, this may lead to lipid perioxidation accumulation (Fig. 1G). In contrast, there was a negative correlation between the GSH/GSSG ratio and ROS levels [30]. The alterations in redox metabolites, particularly the GSH/GSSG ratio, highlight the potential involvement of oxidative stress mechanisms in the context of GPX4 reduction and the development of ferroptosis.

### 2.3 GPX4 spatial distribution in the spinal cord

**Fig. 2:**
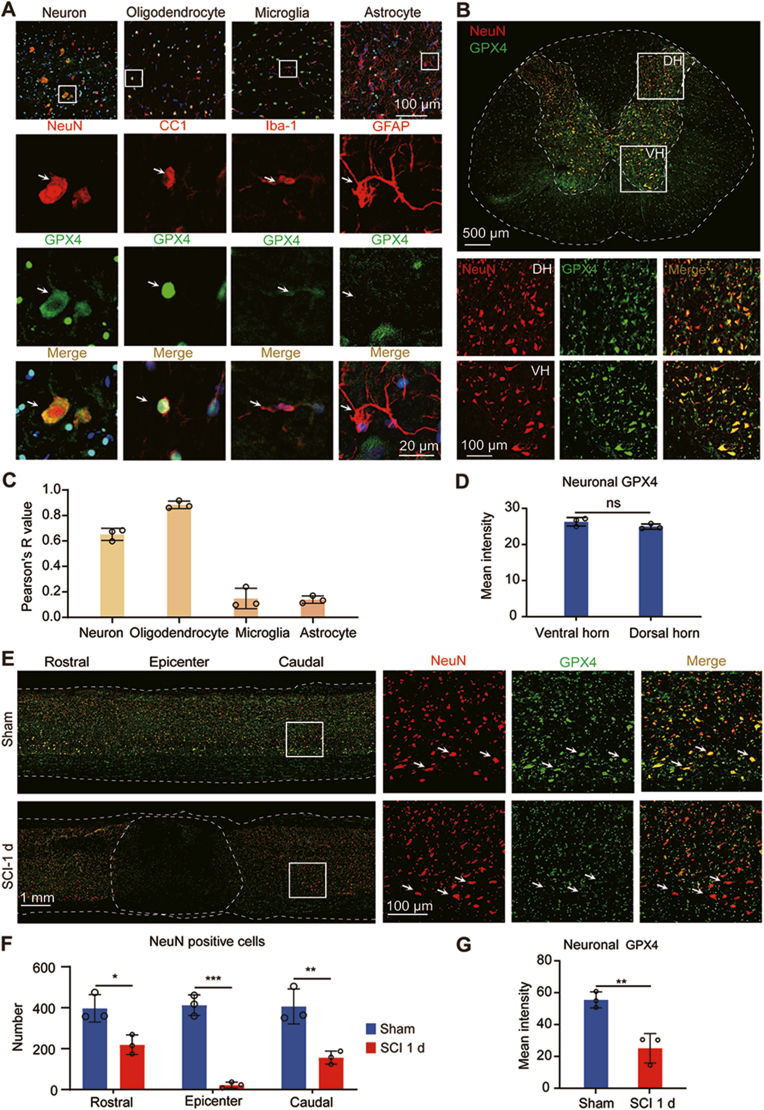
**Spatial expression profile of GPX4 A** Spatial expression of GPX4 in the spinal cord section. Representative images of the intact spinal cord: GPX4 in green, NeuN/CC1/Iba-1/GFAP in red, and DAPI in blue. **B** Coronal section of the intact spinal cord. DH, dorsal horn. VH, ventral horn. **C** Pearson's R value of different cell types. **D** Quantification of dorsal horn and ventral horn neuronal GPX4. **E** GPX4 expression level at 1 d after SCI. GPX4 (green), NeuN (red) and DAPI (blue). **F** Quantification of neuronal GPX4 in the rostral, lesion epicenter and caudal of the spinal cord. **G** Quantifications of neuronal GPX4 at the rostal site of SCI. (n = 3, Data are presented as Mean ± SD, and statistical significance was determined by one-way ANOVA with Tukey's post-hoc test). ∗: p < 0.05, ∗∗: p < 0.01, ∗∗∗: p < 0.001, NS: no significance.

We then examined the spatial distribution of GPX4 expression across different neural cell types within the spinal cord. Immunofluorescence staining in the intact spinal cord revealed distinct patterns of GPX4 expression in various cell populations identified by specific neural cell markers (Fig. 2A). In the intact spinal cord, GPX4 was localized within the neuronal cytoplasm of neurons, as indicated by NeuN labeling. Additionally, GPX4 was observed within the nuclei of oligodendrocytes labeled by CC1. In contrast, GPX4 expression was relatively modest in microglia labeled by Iba-1, and no discernible GPX4 expression was observed in astrocytes labeled by GFAP. The Pearson's R value was used to assess the co-localization between GPX4 and cell-specific markers, indicating that GPX4 is primarily expressed in neurons and oligodendrocytes, with a relatively lower expression in microglia and no detectable expression in astrocytes within the intact spinal cord (Fig. 2C). In coronal sections of the spinal cord, GPX4 expression was predominantly concentrated within the gray matter, with a dotted distribution also present in the white matter (Fig. 2B). The dorsal horn contains somatosensory neurons whereas the ventral horn contains the motor neurons which control the skeletal muscle. Quantification the expression of GPX4 in dorsal horn (DH) and ventral horn (VH) did not see difference (Fig. 2D).

### 2.4 Neuronal GPX4 decrease in the injured spinal cord

Although oligodendrocytes contain abundant GPX4, our previous study found that GPX4 expression in oligodendrocytes remains largely unchanged in the caudal spinal cord segments after SCI [31]. Therefore, we focused on study the changes of GPX4 expression in neurons following SCI using immunofluorescence staining. In the injured spinal cord, a notable decrease in GPX4 expression was observed as early as 1 day post-injury, coinciding with a reduction in neurons at the spinal cord epicenter (Fig. 2E, F,G). This decrease was evident in both GPX4 and neuronal expression levels within the epicenter, attributed to cell death following SCI (Fig. 2 F). In contrast, some neurons were still preserved in the rostral and caudal regions of the spinal cord lesion site, although there was a decrease in neuronal GPX4 expression (Fig. 2 G). This suggests that neurons show a dominant reduction of GPX4 expression in the injured spinal cord. Therefore, our study offers insights into the spatial distribution of GPX4 across various neural cell types in both intact and injured spinal cord tissues, and the significant decrease of GPX4 expression in neurons following SCI offers a new avenue for therapeutics aimed at promoting functional recovery after SCI.

### 2.5 GPX4 inactivation induces neuronal ferroptosis

**Fig. 3:**
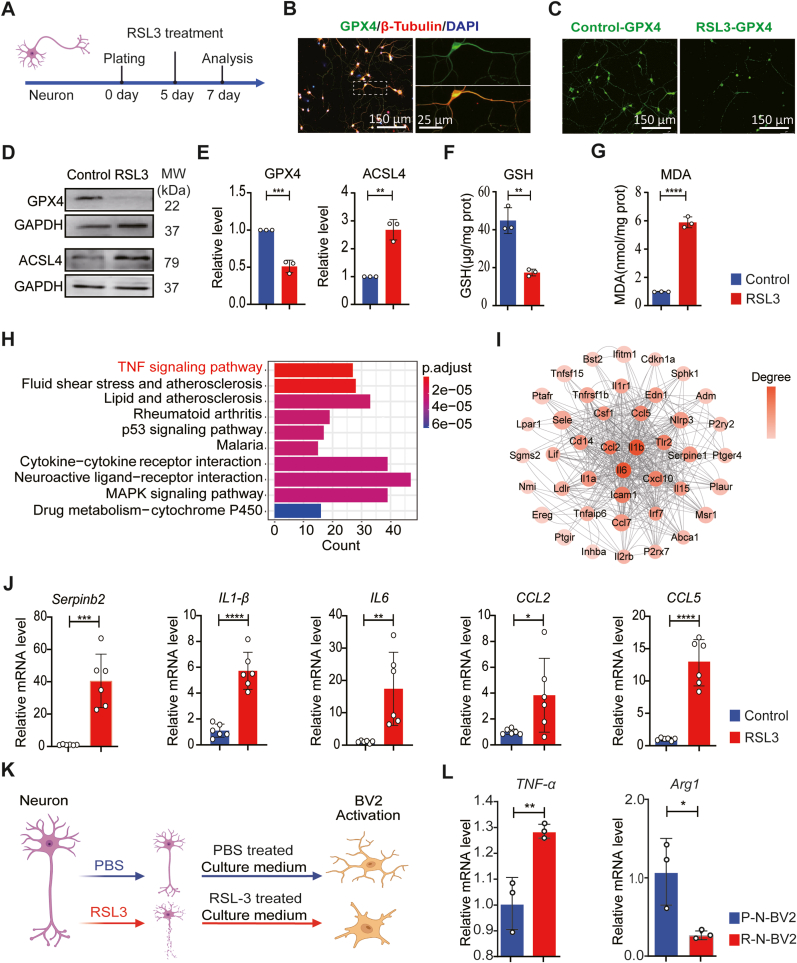
**GPX4 inhibition-induced neuronal ferroptosis is associated with inflammation. A** Experimental design of RSL3-induced ferroptosis in neurons. **B** GPX4 expression in primary neurons. GPX4 (green), β-III-Tubulin (red) and DAPI (blue). **C** GPX4 expression decreases after RSL3 treatment (GPX4 in green). **D** Representative western blot images of GPX4 and ACSL4. **E** Quantification of western blot images (n = 3, Data are presented as Mean ± SD, and statistical significance was determined by unpaired T-TEST). **F** GSH level of RSL3-induced ferroptosis in neurons (n = 3, Data are presented as Mean ± SD, and statistical significance was determined by unpaired T-TEST). **G** MDA level of RSL3-induced ferroptosis in neurons (n = 3, Data are presented as Mean ± SD, and statistical significance was determined by unpaired T-TEST). **H** KEGG of RSL3-induced ferroptosis in neurons. **I** Quantification of key proteins in the DEGs via PPI analysis. **J** Quantification of expression levels of Serpinb2, *IL-1β, IL-6, CCL2, and CCL5* by RT-PCR (n = 6, Data are presented as Mean ± SD, and statistical significance was determined by unpaired T-TEST). **K** Experimental design of microglial activation induced by neuronal ferroptosis. **L** Quantification of TNF-α and Arg-1 after microglial activation by RT-PCR (n = 3, Data are presented as Mean ± SD, and statistical significance was determined by unpaired T-TEST). ∗:p < 0.05, ∗∗: p < 0.01, ∗∗∗: p < 0.001, ∗∗∗∗: p < 0.0001.

In order to understand the role of GPX4 in neurons, we conducted investigations using a primary cortical neuron model of ferroptosis induced by RSL-3, a covalent inhibitor of GPX4 (Fig. 3A). To establish this neuronal ferroptosis model, we first determined the appropriate concentration of RSL-3 for inducing neuronal death. Cell viability was assessed using CCK8 assays after 48 h of RSL-3 induction, resulting in an IC50 value of 3.453 μM (Supplementary Fig. 1A). Consequently, we utilized a concentration of 3.5 μM RSL-3 for subsequent experiments. Immunostaining results for GPX4 and β-III-Tublin indicate that primary neurons express GPX4 within both the cell body and axons (Fig. 3B). Treatment of neurons with RSL-3 resulted in a reduction in neuron numbers and a decrease in GPX4 expression (Fig. 3C), which is consistent with the Western Blot results (Fig. 3D). The expression of acyl-CoA synthetase long-chain family member 4 (ACSL4), a well-established biomarker of ferroptosis capable of converting polyunsaturated fatty acid (PUFA) to peroxidized PUFA-PLs [32], increased in the RSL-3-treated neuron group compared to the control group (Fig. 3D and E). Meanwhile, the levels of the antioxidant glutathione (GSH) notably decreased in RSL-3-treated neurons (Fig. 3F), indicating a redox imbalance following GPX4 inhibition. Additionally, the elevated expression level of malondialdehyde (MDA) in RSL-3-treated neurons confirms the lipid ROS accumulation in cells following GPX4 inhibition (Fig. 3G). Taken together, the GPX4 inhibition through RSL-3 treatment resulted in the induction of ferroptosis within primary neurons cultured *in vitro*. This model provides insights into the molecular mechanisms underlying GPX4's role in neurons and sheds light on the pronounced reduction of GPX4 expression in neurons following SCI.

### 2.6 Neuronal ferroptosis leads to cytokine upregulation and microglial activation

To investigate the correlation between GPX4 inhibition in neurons and subsequent neuroinflammation, we conducted RNA-Seq analysis on both native neurons and neurons treated with RSL-3. The volcano map and heatmap show 1266 upregulated and 281 downregulated genes (Supplementary Figs. 1B and C). Gene Ontology (GO) enrichment analysis demonstrated significant enrichment in genes associated with cytokine production, regulation of inflammatory response, reactive oxygen species, and positive regulation of cell adhesion (Supplementary Fig. 1D). Additionally, KEGG analysis highlighted enrichment in genes related to the TNF signaling pathway, P53 signaling pathway, and MAPK signaling pathway (Fig. 3H). The differentially expressed genes were mainly enriched in the inflammatory response pathway, especially the TNF signaling pathway, which can enhance inflammation. To analyze the relationship between ferroptosis and inflammatory response, the protein–protein interaction network (PPI) analysis was conducted to identify critical proteins among the differentially expressed genes (DEGs) (Fig. 3I). We further validated the major cytokine genes by RT-PCR. The study also identified the upregulation of mRNA of *Serpinb2, CCL2, and CCL5* in primary neurons following GPX4 inhibition (Fig. 3J).

We then explored the induction of microglial activation *in vitro* using the conditioned medium from RSL-3 treated neurons. The medium collected from neurons treated with RSL-3 was used to culture the microglia cell line BV-2 (Fig. 3K). RT-PCR was conducted on the conditioned medium-treated cell line, demonstrating an upregulation in the mRNA level of *TNF-α* (a marker of pro-inflammatory microglia subtype) and a downregulation of *Arg-1* (a marker of anti-inflammatory microglia subtype) (Fig. 3L). Therefore, GPX4 inhibition results in increased cytokine expression in neurons and triggers subsequent microglial activation.

### 2.7 PKUMDL-LC-102 activates GPX4 activity but not GPX4 protein expression level

**Fig. 4:**
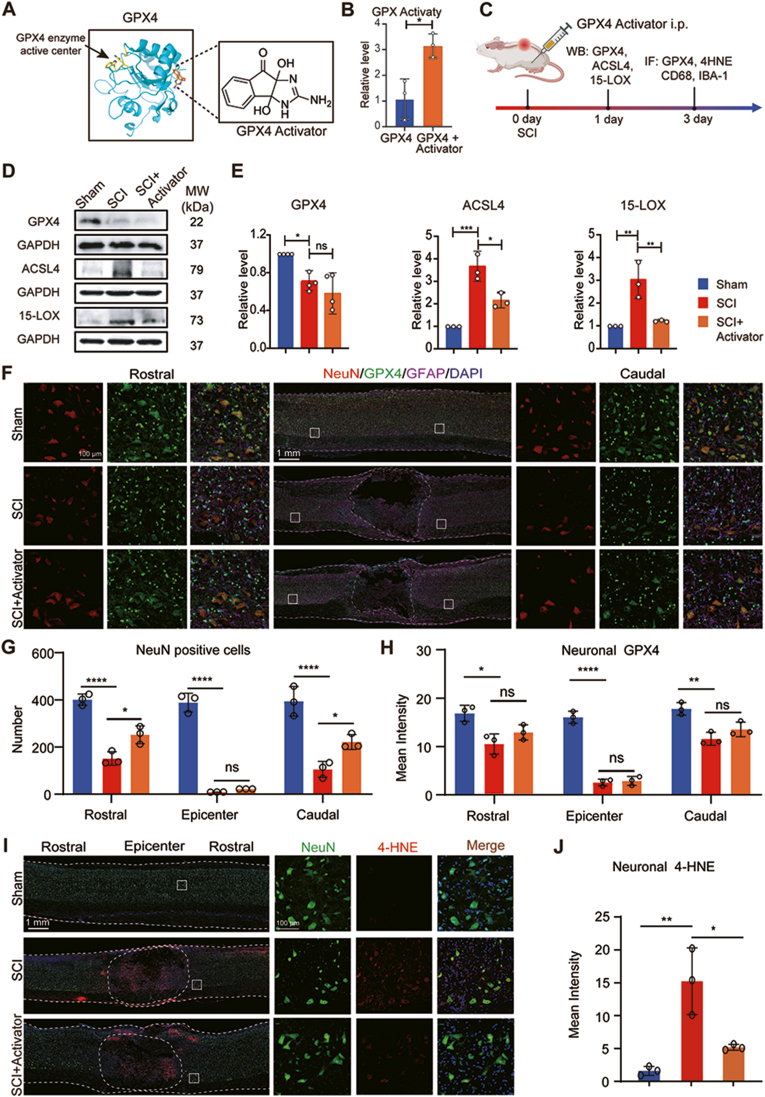
**GPX4 activation regulates ferroptosis. A** Schematic diagram of the GPX4 allosteric activator binds to the opposite side of the GPX4 activity site. Pymol was used to illustrate the predicted binding of the GPX4 activator with the GPX4 crystal structure (Protein Data Bank ID 2OBI). **B** GPX4 activity after adding GPX4 activator. **C** Experimental design of GPX4 activation. **D** Representative western blot images of GPX4, ACSL4, and 15-LOX. **E** Quantification of GPX4, 15-LOX, and ACSL4 protein level at 1 day after SCI (n = 4, Data are presented as Mean ± SD, and statistical significance was determined by one-way ANOVA with Tukey's post-hoc test). **F** Spinal cord sagittal section of the Sham, SCI, and SC + GPX4 activator treatment group. GPX4 in green, NeuN in red, GFAP in magenta, and DAPI in blue. **G** Quantification of the neuron number in the epicenter, rostral, and caudal of the lesion spinal cord segment. **H** Quantifications of neuronal GPX4 of SCI tissue. **I** 4-HNE expression level at 3 day after SCI. NeuN (green), 4-HNE (red), DAPI (blue). **J** Quantifications of neuronal 4-HNE. (n = 3, Data are presented as Mean ± SD, and statistical significance was determined by one-way ANOVA with Tukey's post-hoc test). ∗:p < 0.05, ∗∗: p < 0.01, ∗∗∗: p < 0.001, ∗∗∗∗: p < 0.0001, ns: no significance.

We utilized PKUMDL-LC-102, a small molecule acting as an allosteric GPX4 activator, to investigate the potential neuroprotective effects of GPX4 activation in the context of SCI. PKUMDL-LC-102 activates GPX4 enzymatic activity by binding to the allosteric site opposite the active pocket of GPX4 [26] (Fig. 4A). Biochemical assay demonstrated a threefold increase in GPX4 enzymatic activity upon the addition of the GPX4 activator (Fig. 4B). To explore the impact of GPX4 activation on the ferroptosis pathway in spinal cord tissue, we administered 10 mg/kg of the GPX4 activator intraperitoneally after inducing a contusive SCI in Wistar rats. This dosage was determined through a preliminary experiment indicating that 10 mg/kg is better than 5 mg/kg in exertion neuroprotecion effect in SCI (Supplementary Fig. 1E). Tissue samples from Sham, SCI, and SCI + GPX4 activator groups were collected one day after SCI (Fig. 4C). Western blot results showed that GPX4 expression level decreased in the SCI group compared with the Sham group, but GPX4 activator treatment did not change the expression level of GPX4 in the injured spinal cord compared with the SCI group (Fig. 4D and E). Additionally, Western blot analysis conducted one day after the GPX4 activator administration revealed a downregulating in the expression of both ACSL4 and 15-lipoxygenase (15-LOX) (Fig. 4D and E), which are ferroptosis-specific markers.

### 2.8 GPX4 activation promotes neuronal survival after SCI

Immunofluorescence staining on tissue sections of rats at 3 days post-SCI was conducted to assess the therapeutic impact of the GPX4 activator on ferroptotic neurons. The results revealed a noteworthy effect of the GPX4 activator in protecting neurons in both the rostral and caudal regions of the spinal cord, while there was minimal survival of neurons in the lesion at 3 days post SCI (Fig. 4F and G). GPX4 expression level did not significantly increase following injection the GPX4 activator (Fig. 4H). However, increased lipid peroxidation product 4-Hydroxynonenal (4-HNE) after SCI decreased following treatment with GPX4 activator (Fig. 4I and J). Therefore, these results emphasize the potential of targeted GPX4 activation as a therapeutic strategy to mitigate neuronal loss and potentially promote functional recovery following SCI.

### 2.9 GPX4 activation reduces neuroinflammation after SCI

**Fig. 5:**
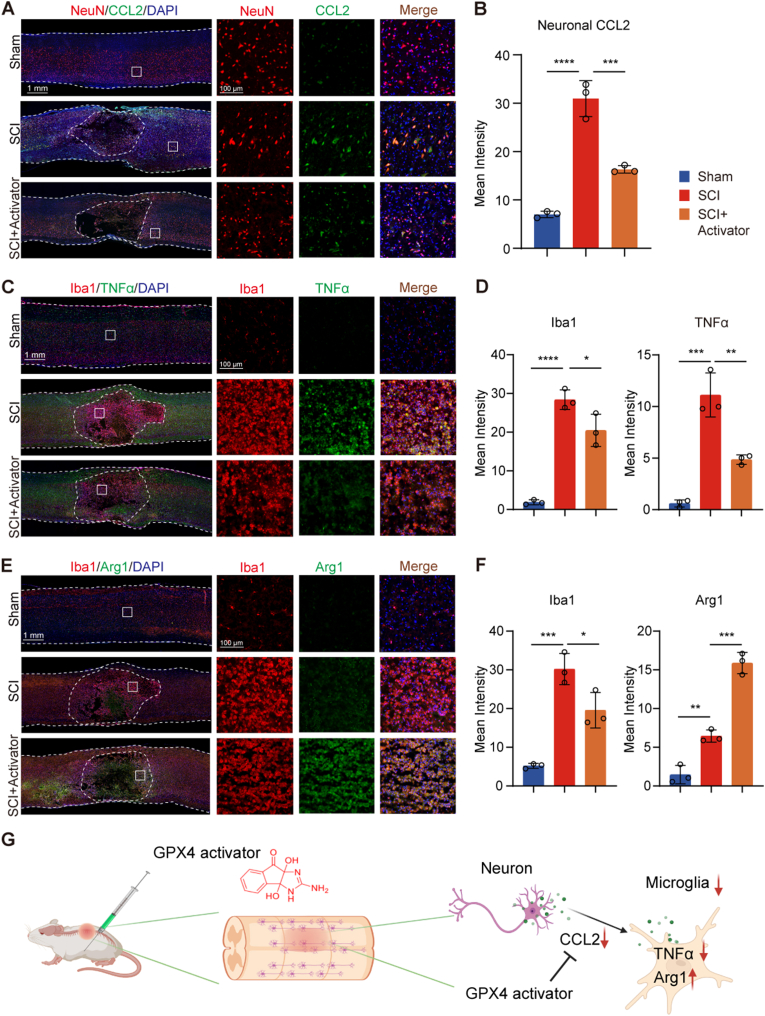
**GPX4 activation regulates neuroinflammation. A** Neuronal CCL2 expression level at 3 day after SCI. NeuN (red), CCL2 (green), DAPI (blue). **B** Quantifications of neuronal CCL2 (n = 3). **C** TNFα expression level in microglia. Iba1 (red), TNFα (green), DAPI (blue). **D** Quantifications of Iba-1 and TNFα. (n = 3). E Arg1 expression level in microglia. Iba1 (red), Arg1 (green), DAPI (blue). **F** Quantifications of Iba-1 and Arg1. (n = 3). G GPX4 activator reduced the secretion of neuronal CCL2 alleviating microglial activation and polarization to pro-inflammatory subtype. Data are expressed as mean ± standard deviation. Statistical significance was determined by one-way ANOVA. ∗: p < 0.05, ∗∗: p < 0.01, ∗∗∗: p < 0.001.

Immunofluorescence staining on tissue sections of rats at 3 days post-SCI was conducted to assess the neuroinflammation of the GPX4 activator. In *vitro* study showed that GPX4 incactivated neuron upregulated the expression of CCL2. Therefore, we explore the expression level of CCL2 in neurons in *vivo*. Consistently, CCL2 in neurons increased after SCI, while the GPX4 activator reduced CCL2 expression in neurons (Fig. 5A and B). Then we further see whether micorglia activation were influenced by GPX4 activator. For microglia, which were labeled by Iba1, there was an increase in the SCI group and a decrease in the SCI + GPX4 activator group (Fig. 5C–F). After GPX4 activator administration, TNFα expression decreased, while Arg1 expression increased in microglia (Fig. 5C–F). These findings indicated that GPX4 activator treatment reduced the secretion of neuronal inflammatory factors, thereby alleviating microglial activation and polarization to pro-inflammatory subtype (Fig. 5G).

### 2.10 GPX4 activation promotes functional recovery after SCI

**Fig. 6:**
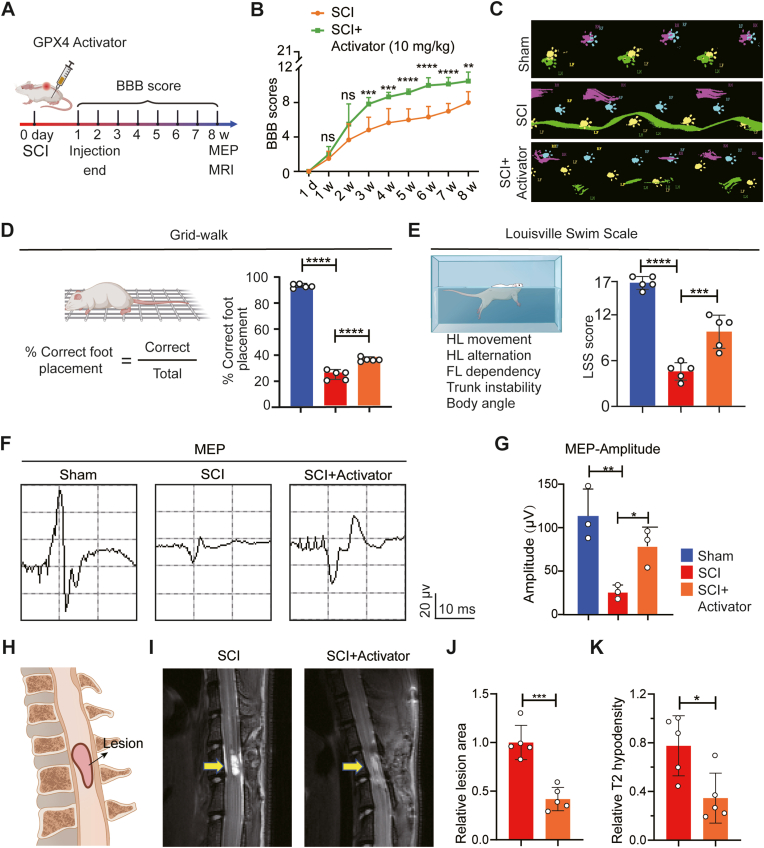
**GPX4 activation promotes functional recovery after SCI. A** Experimental design to detect functional recovery after GPX4 activator administration. **B** BBB scores at different time points after SCI (n = 5, Data are presented as Mean ± SD, and statistical significance was determined by one-way ANOVA with Tukey's post-hoc test). **C** Representative images of footprint at 8 weeks after SCI by catwalk test. **D** Grid-walk test at 8 weeks after SCI (n = 5, Data are presented as Mean ± SD, and statistical significance was determined by one-way ANOVA with Tukey's post-hoc test). **E** Louisville Swim Scale test at 8 weeks after SCI (n = 5, Data are presented as Mean ± SD, and statistical significance was determined by one-way ANOVA with Tukey's post-hoc test). **F** Representative images of electrophysiology of MEP at 8 weeks after SCI. **G** Quantification of the amplitude of MEP (n = 3, Data are presented as Mean ± SD, and statistical significance was determined by one-way ANOVA with Tukey's post-hoc test). **H** Cartoon of lesion shown by MRI. **I** Representative T2WI MRI images at 8 weeks after SCI; **J** Lesion area quantification and **K** relative T2 hypodensity quantification (n = 5, Data are presented as Mean ± SD, and statistical significance was determined by one-way ANOVA with Tukey's post-hoc test). ∗: p < 0.05, ∗∗: p < 0.01, ∗∗∗: p < 0.001, ∗∗∗∗: p < 0.0001, ns: no significance.

We further comprehensively assess the GPX4 activation for promoting functional recovery after SCI (Fig. 6A). The GPX4 activator was administered immediately after SCI and continued for 7 days. Functional recovery was assessed using the Basso, Beattie, and Bresnahan (BBB) scores, which were evaluated weekly for up to 8 weeks after SCI. In the open field hindlimb BBB score test, the experimental group treated with GPX4 activator showed a significantly higher BBB score than the control group without GPX4 activator treatment after 3 weeks post-SCI, indicating an improvement in hindlimb locomotion (Fig. 6B).

Catwalk analysis also demonstrated an improved gait profile following GPX4 activator treatment (Fig. 6C), and the grid walk test also showed an increased correct rate of foot placement at 8 weeks post-injury, indicating improved sensorimotor function (Fig. 6D). Louisville swim scale (LSS) scores in the GPX4 activator-treated group exhibited significant improvement at the 8-week endpoint test (Fig. 6E). Motor Evoked Potentials (MEP) were further monitored to assess the electrophysiological basis of locomotion recovery at 8 weeks post-injury. As a result, the MEP amplitude of the experimental group treated with GPX4 activator was 3-fold higher than the control group without GPX4 activator treatment (Fig. 6F and G). Neuroimaging using Sagittal T2-weighted MRI revealed a reduction in both lesion area and lesion hypodensity for the experimental group treated by GPX4 activator at the 8-week endpoint (Fig. 6H, I, J and K), providing direct evidence of functional recovery after SCI [33]. Therefore, all results, including locomotion, neurological imaging, and electrophysiological data, demonstrate that GPX4 activation can be employed as an effective therapeutic approach to promote functional recovery after SCI.

### 2.11 GPX4 activator pharmacokinetics and safety profile *in vivo*

**Fig. 7:**
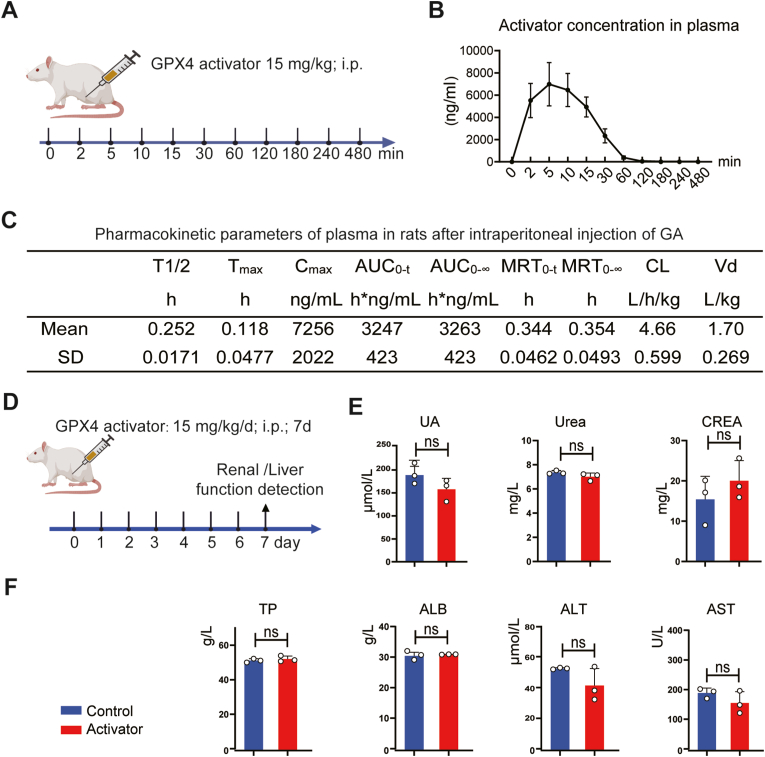
**Pharmacokinetics and safety assessment of GPX4 activator. A** Experimental design of the pharmacokinetics assessment of GPX4 activator. **B** GPX4 activator concentration in plasma at different time points (n = 5, Data are presented as Mean ± SD). **C** Pharmacokinetics data of GPX4 activator (n = 5, Data are presented as Mean ± SD). **D** Experiment design of the safety assessment of GPX4 activator. **E** Renal function assessment (Urea, CREA, and UA) after 7 days' injection of GPX4 activator (n = 3, Data are presented as Mean ± SD, and statistical significance was determined by unpaired T-TEST). **F** Hepatic function (ALT, AST, TP, and ALB) assessment detection after 7 days' injection of GPX4 activator (n = 3, Data are presented as Mean ± SD, and statistical significance was determined by unpaired T-TEST). Abbreviation: T1/2: Terminal Half-life, Tmax: time to reach maximum concentration, Cmax: maximum concentration, AUC(0-t): area under the plasma concentration–time curve from time 0 to last time of quantifiable concentration, AUC(0-∞): area under the plasma concentration–time curve from time 0 extrapolated to infinite time, MRT (0-t): mean residence time–time curve from time 0 to last time of quantifiable concentration, MRT (0-∞): mean residence time–time curve from time 0 extrapolated to infinite time, CL: apparent clearance; Vd: apparent volume of distribution; ALT: Alanine aminotransferase; AST: Aspartate aminotransferase; TP: The total protein; ALB: albumin; UA: Uric acid; CREA: Creatinine. ns: no significance.

The *in vivo* pharmacokinetics and toxicity of the GPX4 activator used in this study were assessed (Fig. 7A). Intact rats were intraperitoneally (i.p.) administered 15 mg/kg of the GPX4 activator, and plasma samples were collected at various time points ranging from 0 to 480 min. The concentration of the GPX4 activator in plasma was measured (Fig. 7B). The findings revealed a rapid increase in the concentration of GPX4 activator in plasma, peaking at over 6000 ng/ml within 5–10 min, followed by a subsequent decrease and return to baseline levels after 120 min (Fig. 7B). Key pharmacokinetic parameters were determined: The terminal half-life (T1/2) of the GPX4 activator was calculated as 0.252 ± 0.0171 h. The time to reach maximum concentration (Tmax) in plasma was determined to be 0.118 ± 0.0477 h with a maximum concentration (Cmax) of 7256 ± 2022 ng. The area under the plasma concentration–time curve from time 0 to the last quantifiable time point (AUC0-t) and the area under the plasma concentration–time curve from time 0 extrapolated to infinite time (AUC0-∞) were 3247 ± 423 and 3263 ± 423 h∗ng/mL, respectively. These values reflect the total amount of the drug reaching the systemic circulation [34]. The mean residence time from time 0 to the last quantifiable time point (MRT0-t) and the mean residence time extrapolated to infinite time (MRT0-me were 0.344 ± 0.0462 h and 0.354 ± 0.0493 h, respectively, insights into the residence time of the GPX4 activator. Drug elimination indicators, clearance (CL) and apparent volume of distribution (Vd), were determined as 4.66 ± 0.599 L/h/kg and 1.70 ± 0.269 L/kg, respectively (Fig. 7C). These pharmacokinetic parameters are critical for understanding the distribution, metabolism, and elimination of the GPX4 activator in the body, offering valuable information for drug development and safety assessment.

To assess potential toxicity, the GPX4 activator was intraperitoneally administrated to intact rats daily for 7 days, covering both the acute and subacute phases of SCI. The effects of the GPX4 activator were evaluated at 7 days after i.p. administration (Fig. 7D). Renal function biomarkers, including uric acid (UA), urea, and creatinine (CREA), were found to be unaffected in the GPX4 activator-treated group compared to the control group (Fig. 7E). Similarly, hepatic function markers, such as total protein (TP), plasma levels of albumin (ALB), alanine aminotransferase (ALT), and aspartate aminotransferase (AST), showed insignificant changes between the control and activator groups (Fig. 7F). These findings indicate that the *in vivo* administration of the GPX4 activator does not induce hepatic or renal toxicity, thus supporting its potential as a therapeutic candidate for functional recovery after SCI without adverse effects on these vital organ systems.

## 3 Discussion

**Fig. 8:**
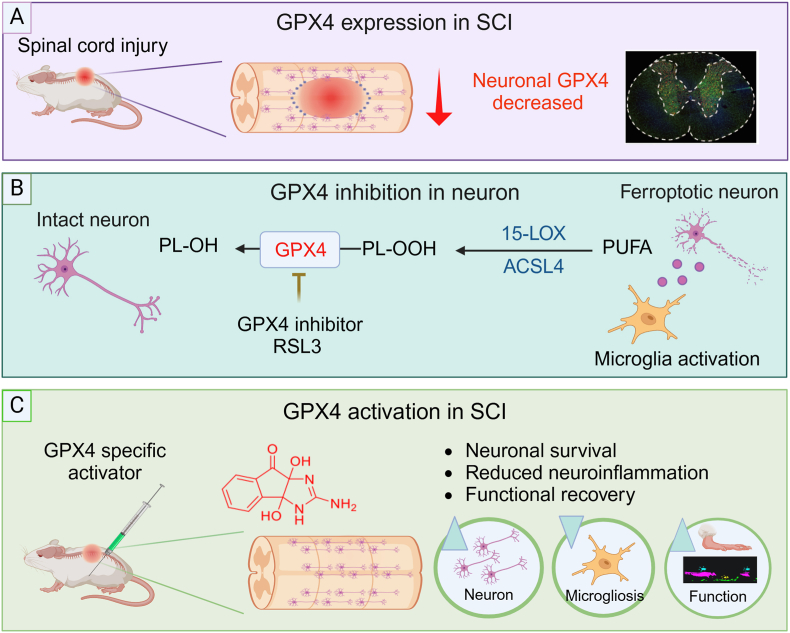
**Schematic illustration of the pivotal role of GPX4 in SCI and the neuroprotection of specific GPX4 activation after SCI. A** GPX4 expression decreases in neurons at acute and subacute stages following SCI. **B** GPX4 inhibition by RSL3 induces ferroptosis in primary neurons *in vitro* and leads to an increase in pro-inflammatory cytokines expression and microgliosis. **C** A specific small molecule GPX4 activator facilitates neuronal survival, reduces microgliosis, and thus promotes functional recovery after SCI.

In this study, we unveil the pivotal role of GPX4 in enhancing the recovery process following SCI through neuronal protection (Fig. 8). Our investigation uncovered the predominant expression of GPX4 within intact spinal cord neurons, with a subsequent downregulation of GPX4 expression observed post-SCI (Fig. 8 A). RNA-seq analysis further revealed a simultaneous upregulation of inflammatory cytokines upon GPX4 inhibition using RSL-3 in primary neurons. Remarkably, we demonstrated that conditioned medium from neurons treated with RSL-3 can induce microglia activation *in vitro* (Fig. 8 B). Moreover, the pharmacological activation of GPX4 using the allosteric activator significantly enhanced functional recovery by inhibiting ferroptosis, preserving neurons, and reducing microgliosis in rats after SCI, which was the first study on specific GPX4 activator applied in an animal model *in vivo* (Fig. 8C). These findings advance our fundamental understanding of GPX4-specific activation as a novel therapeutic strategy for SCI.

GPX4 encompasses three distinct isoforms localized within different subcellular compartments, including mitochondrial, cytosolic, and nuclear isoforms [35]. However, it is important to note that we did not differentiate between these three GPX4 isoforms in our investigation. Specifically, our observations revealed cytosolic expression of GPX4 in neurons and nuclear expression in oligodendrocytes (Fig. 2A). The delineation of these different GPX4 subtypes holds considerable significance for future research endeavors.

The investigation of GPX4 in the context of SCI has suggested potential neuron-microglia crosstalk associated with GPX4. However, further studies are required to explore the specific mechanisms underlying this interaction. The reduction of GPX4 levels in neurons is accompanied by the upregulation of pro-inflammatory cytokine TNF-α in microglia. Further studies are needed to assess the broader impact on inflammatory responses. It is reasonable to infer that chemokines released by ferroptotic neurons may have the potential to recruit and activate microglia. In particular, *Serpinb2* has been reported to link to the enhancement of *CCL2* expression and the regulation of macrophage function in the context of kidney injury [36]. Notably, *CCL2* and *CCL5*, which may be derived from neurons, were identified as potential triggers for microglial activation [37]. In the rat spinal cord we observed the CCL2 upregulation in neurons after SCI, and the reduction by GPX4 activator. CCL2 has been reported to play a role in microglia/macrophage recruitment and polarization in the CNS [38]. Neuronal GPX4 inactivation leads to secreation of proinflammatory cytokines such as CCL2, may further activate local microglia in SCI. In our study, we oberved microglia activation and the proinflammatory polarization of microglia after SCI. And this effect is reversed by GPX4 activator. So both *in vitro* and *in vivo* results supports the neuron-microglia crosstalk which is regulated by GPX4 activity.

In our study, we conducted an assessment of the overall GPX4 levels within the spinal cord and found that GPX4 decreased in the acute phase of SCI. In experimental autoimmune encephalomyelitis (EAE) model, a reduction in neuronal GPX4 was also observed. The mechanism of GPX4 decrease is related with GPX4 degradation by STING induced autophagy [39]. In this study, we did not further explore the mechanism of GPX4 decline but we used a different strategy to elevate the GPX4 activity by a small molecule. Importantly, this research marks the first instance GPX4-activation strategy applied in disease model. Additionally, GPX4 activation operates through a distinct mechanism compared to other well-known ferroptosis inhibitors such as Ferrostatin-1 and Deferoxamine [40,41]. Ferrostatin-1 directly inhibits lipid peroxidation, thereby preventing the accumulation of toxic lipid peroxides, while Deferoxamine functions by chelating iron, reducing the pool of free iron that drives ferroptosis. Since these two classes of ferroptosis inhibitors lack specific target protein, we want to explore a novel kind of ferroptosis inhibitor which has specific target. In our study, the spatial and temporal dynamics of GPX4 is clarified, we use a small molecule compound which drug target is GPX4. The successful application in SCI of this compound provides an opportunity to develop more targeted strategy of GPX4 activator for spinal cord injury repair. In the future, we will use nanoparticle strategy to enhance the delivery efficiency of this drug.

Compared to gene therapy approaches aimed at GPX4 overexpression, the use of a small molecule GPX4 activator offers distinct advantages, particularly in terms of clinical translation. Small molecules are easier to manufacture, can be administered non-invasively, and allow for precise dosage control. Furthermore, small molecules have well-established pathways for regulatory approval, which can accelerate their clinical adoption. This strategy holds great promise for clinical application, given the development and deployment of multiple small molecules to inactivate GPX4 in the context of cancer treatment [42]. This study introduces PKUMDL-LC-102 as an allosteric GPX4 activator, which was previously demonstrated to effectively inhibit ferroptosis *in vitro* [26]. In the SCI rat model, administration of this GPX4 activator promoted functional recovery in locomotion, representing a significant milestone. We observed that GPX4 activator primarily functions by enhancing the enzymatic activity of the existing GPX4 proteins, rather than increasing their expression levels. This allosteric activation may boosted the function of the remaining GPX4 proteins in spinal cord tissues, effectively inhibiting ferroptosis and offering neuroprotection. Furthermore, the safety profile of the activator was rigorously verified, with assessments indicating normal liver and kidney function in treated mice. The pharmacokinetics study revealed a plasma half-life of 0.25 h, highlighting the need for further optimization in future studies.

This research also suggests that applying GPX4 activator may extend beyond SCI to encompass other ferroptosis-related diseases, especially within CNS disorders. Ferroptosis has been widely studied in neurodegenerative diseases [43], traumatic brain injury [44], and EAE [35,45], where GPX4 downregulation has been observed. Ferroptosis contributes to neuronal death and neuroinflammation in these conditions, highlighting its significance in CNS pathology. We discovered a novel anti-ferroptotic effect of GPX4 specific activator *in vivo*. Therefore, insights gained from this study hold great promise for advancing therapeutic interventions.

## 4 Conclusion

Ferroptosis key protein GPX4 decreased in acute and subacute stages following SCI, and most of the reduction occurs in neurons. GPX4 inactivation by RSL3 induced ferroptosis in primary neuron *in vitro*, increased pro-inflammatory cytokine expression, and activated microglia. GPX4 allosteric activator facilitates neuronal survival, reduces microgliosis and promotes functional recovery after SCI.

## 5 Materials and methods

### 5.1 Surgical procedure

A total of 95 Female Wistar rats were used. Rats initially weighing between 180 and 220 g, were procured from Beijing Vital River Laboratory Animal Technology Co. (License No. SCXK (Jing) 2016-0006) in Beijing, China. All animal experiments conducted in this study were ethically approved by the Ethics Committee of the Institute of Radiation Medicine, Chinese Academy of Medical Sciences in Tianjin, China (Approval No. IRM-DWLI-2019110), and were performed following the guidelines outlined in the National Institutes of Health's “Guide for the Care and Use of Laboratory Animals” (NIH Publications no. 85-23, revised 1996). To initiate the experiments, the rats were initially anesthetized using 4–5 % isoflurane (RWD life science, R510-22, Shenzhen, China) and maintained under anesthesia with 2–2.5 % isoflurane. A T10 contusive SCI model was established utilizing the NYU Impactor Model III from the W.M. Keck Center for Collaborative Neuroscience at Rutgers, The State University of New Jersey, USA. This model involved inducing a spinal cord contusion with a 10-g rod at a height of 25 mm. Rats had free access to food and water. Manual bladder expression was performed twice daily until spontaneous urination was restored. In the Sham group, laminectomy was the sole procedure performed without causing any contusion to the spinal cord.

### 5.2 Western blot

The injured spinal cord tissue was collected from the epicenter (0.5 cm in size), which was determined as the central site of the contusion injury. Tissue samples were collected at specific time points post-injury. For the sham group, spinal cord tissue was taken from the corresponding anatomical location. The tissue was lysed using RIPA lysis solution (Beyotime, P0013B, Shanghai, China) on ice for 15 min, followed by centrifugation at 12,000 rpm for 10 min. Protein concentration was determined by measuring absorbance at 562 nm using a BCA kit. Equal protein amounts were loaded onto 12 % Tris–HCl gels. These gels were transferred to PVDF membranes and blocked with 5 % skimmed milk for 60 min. The primary antibodies (GPX4, 1:2500, Abcam, ab125066; ACSL4, 1:2000, Abcam, ab155282; 15-LOX, 1:2000, Abcam, ab244205; GAPDH, Santa Cruz, sc32233, Texas, USA) were incubated overnight at 4 °C. The secondary antibody was incubated at room temperature for 60 min. Finally, the blots were exposed to Immobilon Western HRP Substrate (Millipore, WBKLS0500, Merk, Shanghai, China), and the resulting images were analyzed using Image J.

### 5.3 Immunofluorescence

Rats underwent perfusion with PBS followed by 4 % paraformaldehyde. A 1.5-cm segment of spinal cord tissue from the injured site was post-fixed in 4 % paraformaldehyde overnight. The tissue was subsequently dehydrated using a 30 % sucrose solution. Dehydrated spinal cords were embedded in OCT and then cut into 15 μm-thick sections using a cryostat microtome (Leica, Germany). The sections were rinsed 3 times with PBS and blocked at room temperature for 1 h. Following the blocking step, the sections were incubated with primary antibodies (anti-GPX4, 1:50, Abcam, ab125066; anti-NeuN, 1:200, Abcam, ab104224; anti-CC1, 1:100, Sigma–Aldrich, OP80; anti-4-HNE, 1:50, Invitrogen, MA5-27570; anti-CCL2, 1:100, Servicebio, GB11199; anti-TNFα, 1:200, Abcam, ab183218; anti-Arg1, 1:100, Abcam, ab96183; anti-β-III-Tubulin, 1:500, Abcam, ab78078; anti-GFAP, 1:100, Abcam, ab4674; anti-Iba-1, 1:200, Abcam, ab178846) at 4 °C overnight. After overnight incubation, the sections were washed three times with PBST and then incubated with secondary antibodies (Goat polyclonal Secondary Antibody to Rabbit IgG-H&L (Alexa Fluor® 488), 1:200, Abcam, ab150077; Goat polyclonal Secondary Antibody to Mouse IgG–H&L (Alexa Fluor® 555), 1:200, Abcam, ab150114) at room temperature for 1 h. Following the secondary antibody incubation, the sections were washed three times with PBST and stained with DAPI (Abcam, Ab104139, Cambridge, UK) for 10 min. Finally, images were acquired using a confocal microscope (ZEISS LSM 900, Germany).

### 5.4 Redox metabolites evaluation

Spinal cord tissues, measuring 1 cm in length, were obtained from the injured lesion at 2 days post-SCI. The assessment of redox metabolites was conducted following a previously established method [46]. Chemical standards for GSH (PHR1359), GSSG (V900363), NAD (N4256), NADH (N1511), NADP (N5755), NADPH (N7505), L-Arg (W381918), cysteine (95437), Vitamin C (PHR1008), and L-citrulline (C7629) were procured from Sigma–Aldrich (St. Louis, MO, USA), while GSH-13C215N1 (sc-280747) was acquired from Santa Cruz Biotechnology (Dallas, TX, USA). The spinal cord tissues were homogenized in 75 % methanol containing GSH-13C215N1. Subsequently, the homogenate was mixed with Methyltert-butyl ether (MTBE, 1 ml), followed by adding H_2_O. The resulting mixture was allowed to stand until phase separation occurred. The samples were then subjected to centrifugation at 12,000 g for 10 min at 4 °C, resulting in the separation of the upper organic phase and the lower aqueous phase. The lower phase was transferred directly to a 0.22 μm centrifuge tube before being introduced into the mass spectrometer. Metabolite analysis was conducted using a hybrid triple quadrupole linear ion trap mass spectrometer (5500 QTRAP, AB SCIEX, Foster City, CA), and analytes were detected via multiple reaction monitoring (MRM). The mass spectrometer was operated using Analyst 1.6.1 software (AB SCIEX).

### 5.5 Neuronal ferroptosis *in vitro*

Primary neurons were derived from the embryonic cortex of C57BL/6 mice, which were procured from Beijing Vital River Laboratory Animal Technology Co. These mice were at the gestational age of day 18, following the previously described procedure [47]. Sections of the cerebral cortex were dissected and cut into approximately 1 mm^3^ sections under a stereo microscope. These sections were then treated with papain and DNase for 30 min at 37 °C. After terminating digestion, the cell suspension was centrifuged at 1, 000 rpm for 5 min. Subsequently, the cells were resuspended, and the cell pellet was filtered through a 40 μm cell strainer. The cells were resuspended in a medium composed of DMEM (Invitrogen), 10 % fetal bovine plasma, and 1 % penicillin-streptomycin. These cells were seeded into 96-well (5 × 10^3^/well for cell viability detection), 24-well (3 × 10^4^/well for immunofluorescence staining) plates or 10 cm dish (5 × 10^6^/dish for RNA-sequencing) pre-coated with poly-L-lysine. After a 4-h incubation, the medium was replaced with neuronal medium, which consisted of Neurobasal medium, 2 % B27 supplement (Invitrogen), 1 mM L-glutamine (Invitrogen), and 1 % penicillin and streptomycin (Invitrogen). Half of the neuronal medium was replaced every 3 days until the experiments were completed.

On the 5th day, neurons were treated with the GPX4 inhibitor RSL3 (Selleck Chemicals, S8155) to induce ferroptosis and the sovent is DMSO. Control neurons were treated with PBS. Cell viability was assessed 2 days after RSL3 treatment using the Cell Counting Kit-8 (CCK8, Beyotime, C0038, Shanghai, China), and the optical density (OD) was measured at a wavelength of 450 nm. Based on these results, the IC50 of RSL3 was calculated to induce neuronal ferroptosis, and this concentration was used in subsequent experiments for detecting ferroptosis-related proteins.

### 5.6 MDA and GSH detection

The neurons were cultured on plates for 5 days and subsequently treated with RSL3 for 2 days. After the treatment, cell samples were digested and collected for detection using an MDA assay kit (Solarbio, BC0025, Beijing, China) and a GSH assay kit (SolarBio, BC1175, Beijing, China). All the procedures were conducted in accordance with the manufacturer's instructions for each respective assay kit.

### 5.7 RNA-sequencing and data analysis

Neurons were harvested 48 h after RSL3 treatment, with no media changes during this period. The neurons were collected by digestion and transferred into a centrifuge tube for further analysis. The collected samples were then centrifugated to separate and remove the supernatant. Total RNA was extracted using extraction buffers containing Trizol, phenol and chloroform. Following this, the RNA integrity and the absence of DNA contamination in the samples were assessed through agarose gel electrophoresis. Subsequently, the purity of the RNA was measured using a Nanophotometer spectrophotometer, while the RNA concentration was precisely quantified using a Qubit 2.0 Fluorometer. The RNA integrity was further evaluated using an Agilent 2100 BioAnalyzer, with samples meeting the criteria of concentration ≥750 ng/μl, total RNA ≥2 μg, 28S:18S ratio ≥1, and RIN ≥7.

Library Preparation: The total RNA extracted from the samples with an ensured amount of ≥1 μg was employed for library construction. The library was prepared using Illumina's NebNext® UltrATM RNA Library Prep Kit. First, mRNA was enriched using Oligo (dT) magnetic beads, and then it was randomly fragmented. Subsequently, cDNA fragments of approximately 200 bp in length were selected, amplified through PCR, and purified. High-throughput sequencing was performed to generate sequencing images, which were then converted into sequence data (reads) using Casava base recognition.

Data Analysis: The reference genome index was constructed using the GRCm38 mouse reference genome, and clean reads were aligned using HisAT2 v2.0.5. Differential expression analysis was performed utilizing DESeq2 (https://bioconductor.org/) in R software (version 1.16.1) [48]. The adjusted P-value was determined through DESEQ2 statistical analysis, and a gene with a P-value less than 0.05 was considered differentially expressed between groups. DEGs were identified using a threshold of P-value <0.05 and |log2foldchange | ≥ 2.

Functional Analysis: The GO and KEGG analysis of DEGs was conducted using the DAVID tool (version 6.8, https://david.ncifcrf.gov). Data visualization was performed using ggplot2 package in Rstudio (https://rstudio.com/products/rstudio/).

PPI Analysis: String 11.0 (https://string-db.org/) was employed for protein–protein interaction analysis, while Cytoscape 3.8.2 (https://cytoscape.org/) was used for visual data processing and visualization of the PPI network data.

### 5.8 Quantitative reverse transcription real-time polymerase chain reaction

To isolate RNA from neurons treated with RSL3, the following steps were performed: Neurons were collected and added to 1 ml TRIzol™ (Invitrogen, 15596026, Thermo Fisher, Shanghai, China). Chloroform (0.2 ml per ml of TRIzol™) was added to the mixture, and the solution was vigorously shaken for 15 s. After allowing it to stand for 10 min, the mixture was centrifuged at 12,000 g at 4 °C for 15 min. The top layer, containing total RNA, was carefully collected. To the collected RNA-containing layer, 0.5 ml isopropanol was added, and the mixture was thoroughly mixed. After allowing it to stand for 10 min, the supernatant was discarded. The remaining RNA pellet was washed by adding 1 ml of 75 % ethanol and gently mixed. Centrifugation was performed at 7000 g at 4 °C for 5 min and the supernatant was discarded. The RNA pellet was allowed to air dry briefly, and then 20 μl of sterile water was added to dissolve it. The dissolved RNA was stored in a refrigerator at −80 °C for later use. For quantitative PCR (qPCR), the primers were designed according to Supplementary Table 1 (Table S1), and qPCR results were analyzed using the 2-ΔΔCT method to determine relative gene expression levels.

### 5.9 BV2 cell culture and treatment

The mouse microglia cell line BV2 was obtained from Shanghai Gaining Biological Technology Co., Ltd. These cells were cultured in DMEM/F-12 medium supplemented with 10 % fetal bovine serum (FBS). The medium used to culture neurons was collected and added to BV2 cells for an additional day of culture. This neurons medium likely contained molecules released by neurons undergoing ferroptosis. After this culture period, RT-PCR tests were conducted on the BV2 cells to assess *TNF-α* and *Arg-1* expression.

### 5.10 GPX4 activity assay

To confirm the effect of the GPX4 activator, GPX4 enzyme activity was assessed using the Glutathione Peroxidase Assay Kit (Beyotime, S0056, Shanghai, China) following the manufacturer's instructions. Recombinant GPX4 protein was obtained from Signalway Antibody LLC (Maryland, USA) and dissolved to a concentration of 200 μg/ml. The GPX4 allosteric activator, PKUMDL-LC-102, synthesized by the Lai lab at Peking University [26], was tested at a concentration of 20 μM. This indirect GPX activity determination method relies on decreased nicotinamide adenine dinucleotide phosphate (NAPDH) concentration when NADPH is oxidized to NADP+ in the reaction medium. The reduction in absorbance at 340 nm was monitored to reflect GPX activity. This assay allows for the assessment of how the GPX4 activator impacts the enzymatic activity of GPX4.

### 5.11 Group assignment and drug administration

In the study, Wistar rats were randomly divided into three groups. Sham Group (Sham): This group underwent a sham procedure in which only the T10 vertebral plate was moved without causing any spinal cord injury. Injury Group (SCI): Rats in this group underwent a contusive spinal cord injury at the T10 vertebral level. Injury and GPX4 Activator Intervention Group (SCI + GPX4 Activator): Rats in this group received spinal cord injuries, and they were treated with the GPX4 activator intraperitoneally at a dosage of 10 mg/kg/day, starting immediately after the injury and continuing for seven consecutive days. In some experiment when at 3 days the animal is sacrificed, the drug is given in 3 consecutive days. The GPX4 activator was dissolved in dimethyl sulfoxide (DMSO) at a high concentration of 10 mg/ml. Subsequently, it was diluted with phosphate-buffered saline (PBS).

### 5.12 Behavioral assessment

The behavioral assessment was blindly conducted, ensuring that examiners were unaware of the grouping to minimize bias. BBB score was performed weekly from 1 day to 8 weeks after the surgery [49]. Independent experiments were conducted by three examiners who had studied and mastered the scoring methods. They recorded BBB exercise scores to assess the functional recovery of the lower limbs, using a scale ranging from 0 to 21 points. A score of 0 indicated no observable movement in the hind limbs, and a score of 21 indicated free and adaptive movement in the environment, reflecting a level of normal locomotion before assessment. This assessment method allowed for the objective evaluation of the recovery of lower limb function over time following the surgical interventions without the influence of examiner bias [50].

At 8 weeks after SCI, gait analysis was performed using the Catwalk gait analysis system from Noldus Information Technology B.V, Netherlands. Prior to the analysis, the rats underwent a 3-day training period with two sessions per day to adapt to the walkway and the testing environment. The rats were placed on the Catwalk walkway, which was in a darkened environment. Footprints of the rats were recorded using a high-speed video camera located underneath the walkway. This system allows for the objective assessment of the rats' gait and locomotion patterns, providing valuable insights into their functional recovery after SCI. The hindlimb function was further assessed using Louisville Swim Scale (LSS) Score and Grid–Walk Test at 8 weeks after SCI [33].

The Louisville Swim Scale (LSS) is an 18-point scale, ranging from 0 to 17, designed to assess hindlimb function through the evaluation of five swimming components: forelimb dependency, hindlimb movement, hindlimb alternation, trunk instability, and body position [51]. Rats were placed in a swimming device designed for free swimming at a temperature of 28 °C **for a 1-**min session. Swimming patterns of the rats were recorded using a high-resolution camera. Two independent blinded observers objectively scored the rats based on their performance in these five components.

The grid-walk test was employed to assess the ability of rats to accurately place their hindlimbs on the rungs of a grid during spontaneous exploration. A grid with openings measuring 30 mm × 30 mm was used for this test. Rats were allowed to freely explore the grid for a duration of 3 min. The number of correct hindfoot placements (correctly placed feet on a rung) and the total number of hindfoot placements were counted. The percentage of correct foot placements was calculated as an indicator of hindlimb function. These assessments provided valuable information about the recovery of hindlimb function in the rats following SCI, while the LSS score reflected a broader set of criteria and the grid-walk test focused on precise hindlimb placement during exploration. Both methods were conducted by observers who were blinded to the experimental groups to ensure objectivity in the assessment.

### 5.13 Electrophysiology

At 8 weeks after SCI, MEP was monitored using a neuromonitoring system (YRKJ-G2008, Zhuhai, China) [52]. Rats were anesthetized. The skin was disinfected to ensure a sterile environment for the procedure. A stimulation electrode was positioned under the skin between the ears. A recording electrode was placed in the gastrocnemius muscle of the lower limbs. A stimulation current at 10 mA was applied for MEP. Monitoring MEPs allows for the assessment of motor function by measuring the electrical responses in the muscles, particularly in the gastrocnemius muscle of the lower limbs. MEP monitoring provides valuable information about the functional status of the motor pathways and can be used to evaluate the impact of SCI on motor function and any potential improvements or recovery over time.

### 5.14 MRI

MRI was conducted using a 9.4T MR scanner (BioSpec, 94/30 USR, BRUKER, Germany), and an 8-channel rat receiving coil was employed for signal reception. The MRI parameters were as follows: Echo Time (TE): 23.00 ms; Repetition Time (TR): 690.000 ms; Section Thickness: 1.000 mm; Field of View: 50.000 mm × 50.000 mm; Image Size: 267 × 267 mm. T2-weighted imaging (T2WI) of the rats was performed 8 weeks after SCI while the animals were under anesthesia and at rest. Image analysis was carried out using ParaVision software. The relative lesion area and T2 hypointensity of the spinal cord were obtained according to the sagittal section of the spinal cord.

### 5.15 Pharmacokinetic analysis

In the pharmacokinetics analysis, female Wistar rats weighing 200 g were utilized. The GPX4 activator PKUMDL-LC-102 was prepared, as mentioned earlier. Jugular vein catheterization was performed three days before the experiment to facilitate blood sample collection.

A dose of 15.0 mg/kg of the GPX4 activator was administered via intraperitoneal injection. Blood samples were collected at various time points after the injection, including 0 min, 2 min, 5 min, 10 min, 15 min, 30 min, 1 h, 2 h, 3 h, 4 h, and 8 h.

Plasma was obtained from the collected blood samples through centrifugation. The concentration of the GPX4 activator in the plasma was measured using LC-MS/MS with the following equipment: LC System: SHIMADZU LC-20AD, Mass Spectrometer: AB 4000 (Analyst 1.6.2), A Kinetex® C18 2.6 μm 3 × 100 mm column was used for chromatography.

The major pharmacokinetic parameters were calculated using WinNonlin 7.0. These parameters include: Half-life (T1/2), Time to maximum concentration (Tmax), Maximum concentration (Cmax), Area under the concentration–time curve (AUC) from time 0 to the last measurable concentration (AUC(0-t)), Area under the concentration–time curve from time 0 to infinity (AUCINF), Volume of distribution (Vd), Clearance (CL), and Mean residence time (MRT). These calculations provide essential information about the absorption, distribution, metabolism, and elimination of the GPX4 activator in the body, aiding in understanding its pharmacokinetic profile.

### 5.16 Safety assessment

Safety assessment was conducted after 7 days of administering different concentrations of the GPX4 activator (15 mg/kg/day) to Wistar rats. Blood samples were collected via the inner canthus for this assessment, which focused on evaluating liver and kidney function. The assessment included the following parameters, which were measured using an AU5800 automatic biochemical analyzer from Beckman Coulter, Inc, Tokyo, Japan: Alanine Aminotransferase (ALT): ALT is an enzyme found in the liver, and its levels can indicate liver health. Aspartate Aminotransferase (AST): AST is another enzyme primarily located in the liver and can provide insights into liver function. Total Protein (TP): Total protein levels in the blood can indicate overall protein status in the body. Albumin (ALB): Albumin is a protein produced by the liver, and its levels can reflect liver function and overall protein balance. Uric Acid (UA): Uric acid is a waste product in the blood, and elevated levels can be associated with kidney or metabolic issues. Urea: Urea levels can be used to assess kidney function, as the kidneys play a key role in filtering and excreting urea. Creatinine (CREA): Creatinine is a waste product from muscle metabolism and is filtered by the kidneys. Elevated levels can indicate kidney dysfunction. These biochemical parameters were analyzed to evaluate the safety profile of the GPX4 activator and to ensure that it had no adverse effects on liver and kidney function in the rats.

### 5.17 Statistics

GraphPad Prism 9 software (San Diego, CA) was used for statistical analysis. Prior to selecting statistical methods, normality tests were performed on all datasets to determine whether parametric or nonparametric methods were appropriate. For normally distributed data, a T-TEST is used to compare the two groups. One-way analysis of variance (ANOVA) was used for multi-group comparisons, followed by Tukey's post-hoc test for multiple comparisons. Behavioral scores were evaluated using repeatable two-way ANOVA and corrected by Tukey. All data were shown in the form of mean standard deviation. P < 0.05 indicates that the difference is statistically significant.

## Author contributions

X.L, Y.P, and B.F. contributed equally to this work. X.L.,Y.P., and B.F. co-designed the study, conducted experiments, analyzed and interpreted the data, co-made the figures, and co-wrote the manuscript. Y.P., J.Z., and X.L. conducted SCI surgery and western blotting. B.F. assisted in neuron cultures and provided RNA sequencing data. C.Z. and T.Z. performed analyses. Y.L. assisted with small molecule biosafety study. X.Z. performed LC-MS/MS analysis of redox metabolites. Y.L. and L.L. assisted in GPX4 activator studies. X.W. and G.N. co-supervised the project and contributed to experimental design, interpretation, and manuscript editing. X.Y., W.L., and S.F. supervised the project, guided experimental design and interpretation, co-made the figures, and co-wrote the manuscript. These authors declare that they have no competing interests.

## Availability of data and materials

The data in this study were shown in the main text and supplementary. The data that support the findings of this study are available from the corresponding author upon reasonable request.

## Declaration of competing interest

X.L, Y.P, and B.F. contributed equally to this work. X.L.,Y.P., and B.F. co-designed the study, conducted experiments, analyzed and interpreted the data, co-made the figures, and co-wrote the manuscript. Y.P., J.Z., and X.L. conducted SCI surgery and western blotting. B.F. assisted in neuron cultures and provided RNA sequencing data. C.Z. and T.Z. performed analyses. Y.L. assisted with small molecule biosafety study. X.Z. performed LC-MS/MS analysis of redox metabolites. Y.L. and L.L. assisted in GPX4 activator studies. X.W. and G.N. co-supervised the project and contributed to experimental design, interpretation, and manuscript editing. X.Y., W.L., and S.F. supervised the project, guided experimental design and interpretation, co-made the figures, and co-wrote the manuscript. All authors declare that we have no known competing financial interests or personal relationships that could have appeared to influence the work reported in this paper. This declaration serves to demonstrate our commitment to scientific ethics and to ensure the trustworthiness of our research findings.

## Figures and Tables

**figs1:**
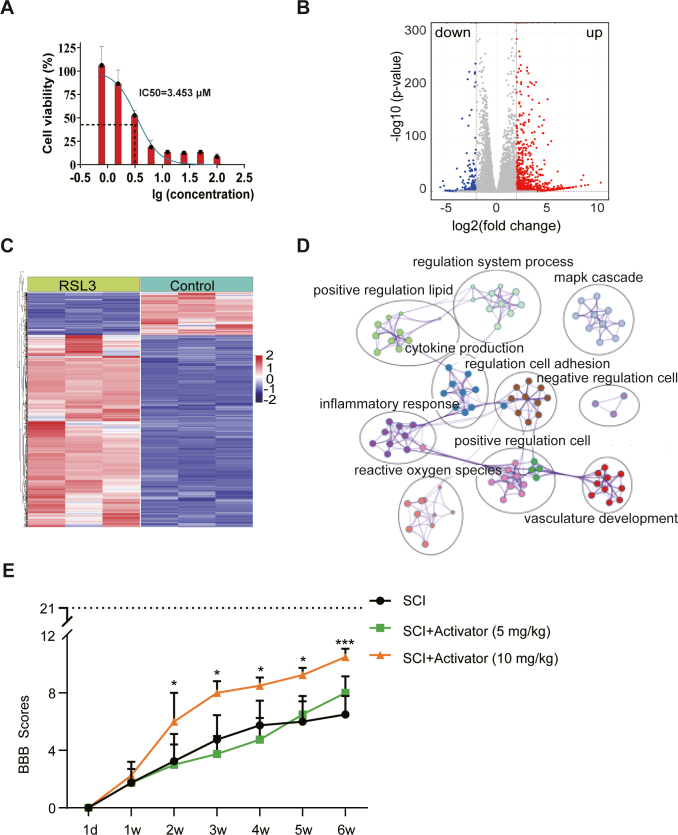
GPX4 inhibitor RSL3 induces neuronal ferroptosis model in vitro. A IC50 of GPX4 inhibitor RSL3 in neuronal ferroptosis model in vitro. Neurons were treated with different concentrations of RSL3 for 48 hours and cell viability was detected using CCK8 (n=5, Data are presented as Mean ± SD). B Volcano map of the DEGS of RSL3-induced ferroptosis in neurons. C Heatmap of DEG of RSL3-treated and untreated neurons. D GO enrichment analysis was conducted to clarify gene function. E BBB scores for different dosages of GPX4 activator after SCI (n=5, Data are presented as Mean ± SD, and statistical significance was determined by one-way ANOVA with Tukey’s post-hoc test).
