# Supplementary material for: GPX4 activator enhances neuroprotection and functional recovery in spinal cord injury
Source: J Orthop Translat. 2025 May 7;52:344–59. doi: 10.1016/j.jot.2025.03.013 (PMC12143174; doi:10.1016/j.jot.2025.03.013)
Supplement: Multimedia component 1 [file mmc1.docx]

**Supplementary Table 1** Primers for qPCR

| Target gene | Description | Sequence (5’>3’) |
| --- | --- | --- |
| Gapdh | Forward Primer | GGTGAAGGTCGGTGTGAACG |
|  | Reverse Primer | CTCGCTCCTGGAAGATGGTG |
| Serpinb2 | Forward Primer  Reverse Primer | GCCCTCAATCTCCTTAAGCA  TCAGCACATTCAAGGAAGTCCA |
| Il-1β | Forward Primer | GCCACCTTTTGACAGTGATGAG |
|  | Reverse Primer | AAGGTCCACGGGAAAGACAC |
| Il-6 | Forward Primer | GGATATAATCAGGAAATTTGCCTA |
|  | Reverse Primer | TTCCAAGAAACCATCTGGCTA |
| Ccl2 | Forward Primer  Reverse Primer | CTCTCACTGAAGCCAGCTC  ATTAGCTTCAGATTTACGGGTCA |
| Ccl5 | Forward Primer | GCTCCAATCTTGCAGTCGTG |
|  | Reverse Primer | AGAGCAAGCAATGACAGGGA |
| Tnfα | Forward Primer  Reverse Primer | GATTCTTCCCTGAGGTGCAA  TCAGCGTTATTAAGACAATTGGGT |
| Arg-1 | Forward Primer  Reverse Primer | AAGAAATTTACAAGACAGGGCTC  ATACATCTTTTGAACAGCGTGGA |
